# Seed dormancy types and germination response of 15 plant species in temperate montane peatlands

**DOI:** 10.1002/ece3.11671

**Published:** 2024-07-01

**Authors:** Jian‐Yi Wang, Zhao‐Jun Bu, Peter Poschlod, Shuayib Yusup, Jia‐Qi Zhang, Zheng‐Xiang Zhang

**Affiliations:** ^1^ Key Laboratory of Geographical Processes and Ecological Security in Changbai Mountains, Ministry of Education, School of Geographical Sciences Northeast Normal University Changchun China; ^2^ State Environmental Protection Key Laboratory of Wetland Ecology and Vegetation Restoration Institute for Peat and Mire Research, Northeast Normal University Changchun China; ^3^ Jilin Provincial Key Laboratory for Wetland Ecological Processes and Environmental Change in the Changbai Mountains Changchun China; ^4^ Institute of Plant Sciences, Ecology and Conservation Biology University of Regensburg Regensburg Germany

**Keywords:** Changbai Mountains, cold stratification, germination response, peatlands, physiological dormancy

## Abstract

Despite their crucial role in determining the fate of seeds, the type and breaking mode of seed dormancy in peatland plants in temperate Asia with a continental monsoon climate are rarely known. Fifteen common peatland plant species were used to test their seed germination response to various dormancy‐breaking treatments, including dry storage (D), gibberellin acid soaking (GA), cold stratification (CS), warm followed cold stratification (WCS), GA soaking + cold stratification (GA + CS) and GA soaking + warm followed cold stratification (GA + WCS). Germination experiment, viability and imbibition test, and morphological observation of embryos were conducted. Of the 15 species, nine showed physiological dormancy (PD), with non‐deep PD being the dominant type. Four species, *Angelica pubescens*, *Cicuta virosa*, *Iris laevigata*, and *Iris setosa* exhibited morphophysiological dormancy. Two species, *Lycopus uniflorus* and *Spiraea salicifolia*, demonstrated nondormancy. Overall, the effect hierarchy of dormancy‐breaking is: CS > GA > WCS > GA + CS > D > GA + WCS. Principal component analysis demonstrated that seed traits, including embryo length: seed length ratio, seed size, and monocot/eudicot divergence, are more likely to influence seed dormancy than environmental factors. Our study suggests that nearly 90% of the tested peatland plant species in the Changbai Mountains demonstrated seed dormancy, and seed traits (e.g. embryo‐to‐seed ratio and seed size) and abiotic environmental factors (e.g. pH and temperature seasonality) are related to germination behavior, suggesting seed dormancy being a common adaptation strategy for the peatland plants in the temperate montane environment.

## Graphical abstract

Nearly, 90% of the tested peatland plant species demonstrate seed dormancy, mainly physiological dormancy.Seed traits, are more likely to influence seed dormancy than environmental factors.

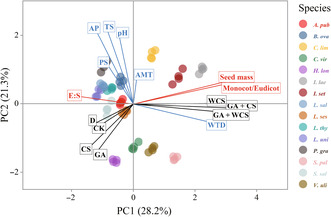

## 1 INTRODUCTION

Seed dormancy is an important adaptation strategy for numerous plant species to coordinate germination and establishment with the environment (Baskin & Baskin, 2014; Thompson & Grime, 1979). Generally, dormancy is controlled by both internal and external factors, and is one of the important life history features of plants, enabling them to cope with the cyclic and fluctuating environmental conditions (Finch‐Savage & Leubner‐Metzger, 2006). In harsh environment, to reduce the probability of sexual regeneration failure, plants tend to employ seed dormancy to prevent simultaneous germination of all seeds (Long et al., 2015). This delay in germination may ensure the persistence of seed banks and continuous regeneration of the plant community (Saatkamp et al., 2014). In the region of temperate Asia with continental monsoon climate, seasonality (namely, seasonal fluctuation) is rather strong and the frigid weather in early and growing seasons is more harsh than in other temperate, subtropical, and tropical regions (Cheng et al., 2017). However, the type and breaking mode of seed dormancy in the region are rarely known.

The means of dormancy releasing can vary depending on the type of seed dormancy. For seeds with PY, scarification, or high‐temperature treatments are required to break the dormancy (Lamont & Pausas, 2023). For PD, under the dual control of germination accelerator (GA) and germination inhibitor (ABA), seeds present a dormancy continuum, that is, states between dormancy and nondormancy (Footitt et al., 2011). Environment factors (temperature, light, oxygen, water, and chemicals) can cause changes in dormant states, and dormancy (non‐deep PD) can be broken by gibberellic acid soaking or environmental signals, such as cold and wet, or warm and wet weather (Baskin & Baskin, 2004). Seeds with MD have an undeveloped or undifferentiated embryo at seed maturity, and the dormancy can be released without pretreatment or with dry storage as the embryo grows (Baskin & Baskin, 2004). Seeds with MPD, which has both morphological and physiological dormancy characteristics and is sensitive to environmental change, can be broken by stratification (warm, cold, or alternating temperature) or GA. Seeds with combinational dormancy (PY + PD), exhibiting both physical and physiological dormancy, can be broken by scarification followed by stratification (Baskin & Baskin, 2014).

The effectiveness of dormancy releasing is means‐dependent. Dry storage, as a means to break seed PY (Gama‐Arachchige et al., 2012; Jayasuriya et al., 2008), is an effective way to break intermediate PD and MPD of seeds (Baskin & Baskin, 2020; Finch‐Savage & Leubner‐Metzger, 2006). Compared with dry storage, cold stratification (CS), in which seeds are exposed to moist and cold conditions (0–10°C) for at least 4–6 weeks (Merritt et al., 2007), is considered to be the much more practical way to break PD of the seeds. In temperate regions, CS is usually required for seeds to break PD in spring (Baskin & Baskin, 2014). A lot of studies show that GA can partially replace CS in breaking PD/MPD (e.g. Baskin & Baskin, 2014; Hao et al., 2014; Herranz et al., 2010; Zhang et al., 2019), but it cannot break deep PD (Baskin et al., 2005) and deep complex MPD (Phartyal et al., 2009; Walck & Hidayati, 2004). Therefore, the effectiveness of GA is generally lower than that of CS (Chien et al., 2011; Li et al., 2022; Wang et al., 2017). In temperate and boreal regions, warm stratification (WS) is usually required for the seeds with PD/MPD to geminate in autumn and WS followed by CS (hereafter called WCS) is needed for the seeds to break PD/MPD in spring (Baskin & Baskin, 2014). It has been reported that WCS greatly enhances seed germination by well‐breaking PD/MPD compared with WS or CS alone (Baskin & Baskin, 1989; Liu et al., 2023) and WS followed GA is effective in breaking PD (Chen et al., 2008, 2022). A combined treatment of GA and CS has been found to result in a greater seed germination than either treatment alone (Hashemirad et al., 2023; Li et al., 2022).

Seed size/mass (Gioria et al., 2020; Hashemirad et al., 2023; Rees, 1993; Shipley & Parent, 1991; Thompson & Grime, 1979), phylogeny (Rosbakh et al., 2019; Rubio de Casas et al., 2017; Scholten et al., 2009) and environmental factors especially climate factors (Carta et al., 2022; Rosbakh et al., 2023) were proved to jointly determine seed dormancy. Seed size/mass is a crucial seed trait. Numerous studies indicate that small‐seeded species have a higher proportion of dormancy than large‐seeded counterparts (Gioria et al., 2020; Rees, 1993, 1996; Thompson & Grime, 1979). Large‐seeded species possess an advantage in seedling establishment due to more nutrient supply in resource‐limited conditions. This exempts them from dormancy maintenance and maximizing their growth before adverse conditions occur (Rees, 1996). Conversely, small‐seeded species are less competitive and often require a vegetation gap after disturbance to accomplish seedling establishment. Considering plant phylogeny, PD is very common in some families, such as Cyperaceae (Rosbakh et al., 2019;  Schütz, 1997) and Poaceae (Fernández‐Pascual et al., 2022). Morphological dormancy (MD) and/or morphophysiological dormancy (MPD) are prevalent in Apiaceae, and MPD was also observed in various *Iris* and *Lobelia* species (Baskin & Baskin, 2014). Physical dormancy (PY) predominates in Fabaceae (Rubio de Casas et al., 2017). At a large scale, climatic factors such as seasonality, average annual temperature, and annual precipitation are associated with seed dormancy types (Rosbakh et al., 2023; Zhang, Liu, et al., 2022). For example, PY is more common in the area with strong seasonality in both temperature and precipitation; PD is predominated in relatively dry area with high‐emperature seasonality; and nondormancy is associated with stable, warm and humid climates (Baskin & Baskin, 2014; Jurado & Flores, 2005; Rosbakh et al., 2023). Seeds of many peatland plants can exhibit dormancy, predominantly PD (Fernández‐Pascual, 2016; Fernández‐Pascual et al., 2013; Jensen, 2004; Poschlod, 1990;  2019 Rosbakh et al., 2020; Schütz, 1997). For example, from 20 peatland species (13 raised bog or facultatively raised bog species, 15 fen or facultatively fen species) in Southeast‐Germany, 13 species showed a physiological dormancy (Poschlod, 1990). Six *Carex* species in northern Germany showed a seasonal dormancy cycle (Schütz, 1997). Fen plants were found to commonly show PD of seeds and CS and even WS effectively increased seed germination in 15 tested species in Spain (Fernández‐Pascual et al., 2013). A further study on 34 peatland species showed that the seeds of all species exhibited PD at dispersal, and 28 of the 34 species showed conditional type 2 non‐deep PD (Fernández‐Pascual, 2016).

Northern peatlands are rich in ecological gradients, such as water table depth and pH (Rydin & Jeglum, 2013; Wheeler & Proctor, 2000). Along the gradient of water table depth and pH, peatlands differentiated as hummocks and hollows and bogs and fens, respectively. The characterized ecological gradients affect ecological processes in peatlands (Rydin & Jeglum, 2013). For example, the germination of seeds (Fernández‐Pascual, 2016; Liu et al., 2005) and spores (Fan et al., 2023; Feng et al., 2018; Sundberg & Rydin, 2000) is strongly depressed by hypoxia in hollows with low water table depth. In *Sphagnum* dwelling peatlands, the strong acidity due to cation exchange by *Sphagnum* is believed as the main mechanism to suppress seed germination and then vascular plant colonization (van Breemen, 1995) even though spore germinability (represented by germination percentage) decreases with pH in *Sphagnum* (Feng et al., 2018). We, however, know rather less about seed dormancy type and even germination characteristics of peatland plants in temperate Asia with terrestrial monsoon climate, one of the main distribution areas for peatlands in the world.

To address the knowledge gap, we selected 15 vascular plant species collected from five peatlands in the Changbai Mountains of northeastern China, and tried to test the hypotheses through various means of dormancy breaking. Specifically, we hypothesized that: (1) most of the tested peatland plants would exhibit PD in seeds, with non‐deep PD being predominant; (2) for the effectiveness of dormancy breaking, combined treatments would be better than single treatments, while CS would be better than GA and dry storage; (3) seed size would negatively be correlated with germination percentage; and (4) The germinability would be related with maternal environmental characteristics, namely, positively correlated with water table depth and negatively correlated with pH.

## 2 MATERIALS AND METHODS

### 2.1 Study area

**FIGURE 1:**
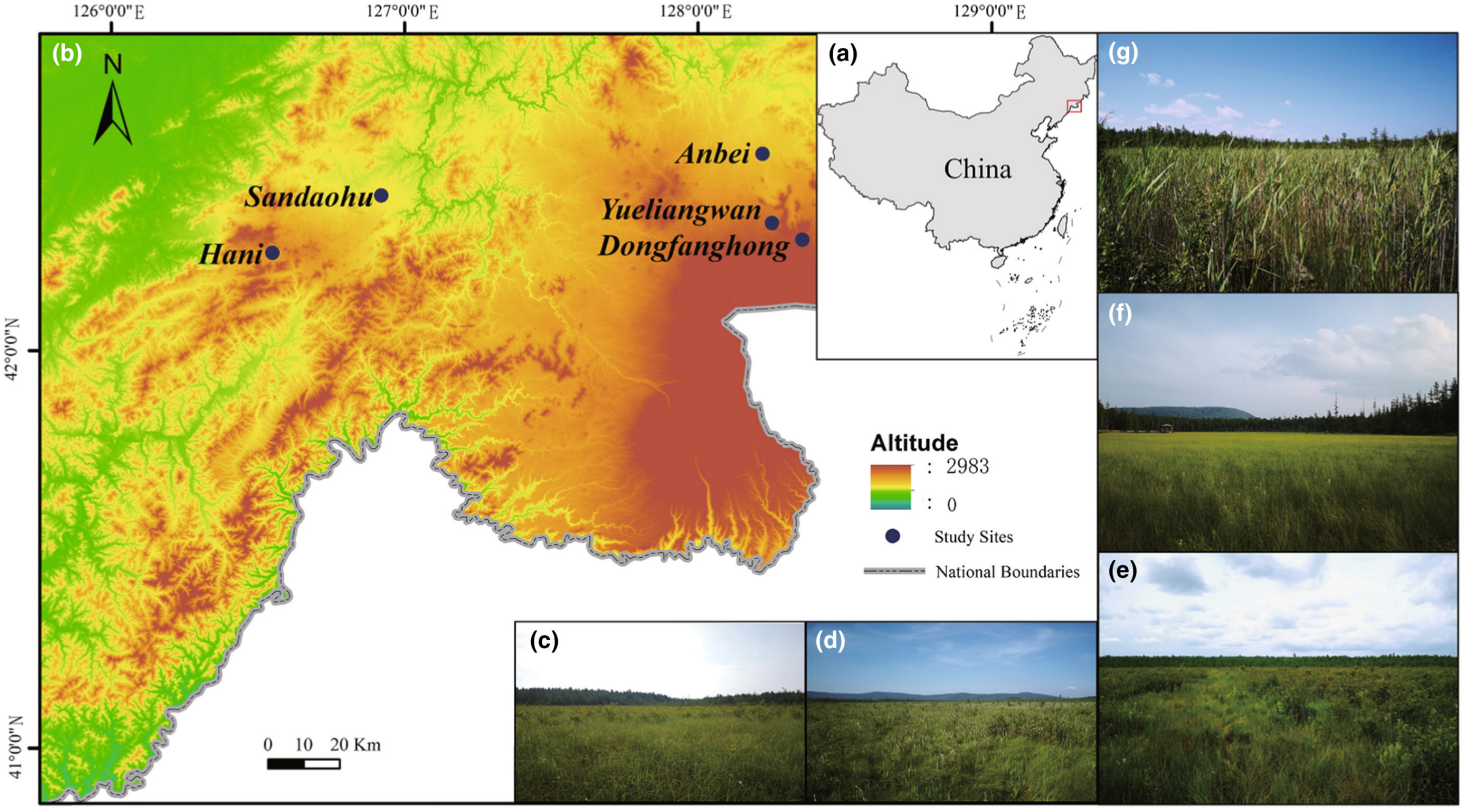
Maps showing the location of the study region in China (a) and study sites in the Changbai Mountains, northeastern China (b) and landscape photographs of Hani (c), Sandaohu (d), Anbei (e), Yueliangwan (f), and Dongfanghong (g) peatlands.

The Changbai Mountains is one of the main distribution regions of peatlands in China, covering a geographical span of 38°46′–47°30′ N and 121°08′–130°20′ E. The highest elevation in the area is 2691 m, and the lowest elevation is 410 m. Under the control of the East Asian monsoon, the study area is characterized by a mid‐temperate continental monsoon‐type mountain climate with four distinct seasons. In our study area (Figure 1), the mean annual temperature ranges from −4 to 9°C, and the annual precipitation ranges from 679 to 862 mm (data sources: WorldClim database). The growing season is from May to September, and the freezing period is from October to April. In peatlands, the vegetation is plant species rich and usually include diverse bryophytes, herbs, dwarf shrubs, and sparse trees. Among them, *Sphagnum* spp., *Carex* spp., *Betula ovalifolia* Rupr., *Vaccinium uliginosum* L., and *Larix olgensis* A. Henry are common (Bu et al., 2011).

### 2.2 Seed collection

Fifteen species including three woody species and 12 herbaceous species from four habitats (Table 1) were chosen as the study species since they are common in the peatlands in the Changbai Mountains and their seed amounts are great enough for experiment use. During middle August–late September, 2020–2021, their seeds were collected from five peatlands (Figure 1). The collected seeds were air‐dried at laboratory conditions of RH 46% and 22°C until they reached a constant weight and were mixed thoroughly to ensure uniform seed quality (De Vitis et al., 2019). Only intact seeds without empty shells or mildew were used in the experiment. The collected fresh seeds for each species were air‐dried for 1 week and then were divided into eight groups, with 100 seeds in each group. Each group of seeds was weighed using a Sartorius analytical balance (BS224S, 0.0001 g).

**TABLE 1:** Information on the study species, seed mass, seed collection sites and habitats.

Species	Abbr.	Life form	Seed mass/g	Site	Habitat	WTD/cm	pH
*Angelica pubescens*	*A. pub*	Forb	2.231 (0.000)	H	F‐Hu	12.7 (3.37)	6.13 (0.160)
*Betula ovalifolia*	*B. ova*	Dwarf shrub	0.628 (0.000)	H	F‐Hu	12.7 (3.37)	6.13 (0.160)
*Carex limosa*	*C. lim*	Graminoid	1.161 (0.001)	S	O‐Ho	5.0 (3.27)	6.08 (0.052)
*Cicuta virosa*	*C. vir*	Forb	0.788 (0.000)	A	F‐Hu	9.0 (5.97)	5.35 (0.093)
*Hypericum longistylum*	*H. lon*	Forb	0.131 (0.001)	A	F‐Hu	9.0 (5.97)	5.35 (0.093)
*Iris laevigata*	*I. lae*	Forb	20.908 (0.000)	H	O‐Ho	19.1 (3.65)	6.03 (0.072)
*Iris setosa*	*I. set*	Forb	6.923 (0.000)	H	O‐Ho	19.1 (3.65)	6.03 (0.072)
*Lobelia sessilifolia*	*L. ses*	Forb	0.190 (0.000)	H	O‐Hu	19.1 (3.65)	6.03 (0.072)
*Lycopus uniflorus*	*L. uni*	Forb	0.171 (0.001)	S	F‐Hu	5.0 (0.82)	5.85 (0.081)
*Lysimachia thyrsiflora*	*L. thy*	Forb	0.326 (0.000)	S	O‐Hu	5.0 (3.27)	6.08 (0.052)
*Lythrum salicaria*	*L. sal*	Forb	0.035 (0.000)	S	O‐Ho	5.0 (3.27)	6.08 (0.052)
*Pedicularis grandiflora*	*P. gra*	Forb	0.968 (0.000)	S	O‐Ho	5.0 (3.27)	6.08 (0.052)
*Scheuchzeria palustris*	*S. pal*	Graminoid	5.231 (0.000)	Y	O‐Ho	15.9 (3.66)	5.50 (0.130)
*Spiraea salicifolia*	*S. sal*	Dwarf shrub	0.062 (0.000)	H	F‐Hu	12.7 (3.37)	6.13 (0.160)
*Vaccinium uliginosum*	*V. uli*	Dwarf shrub	0.228 (0.000)	D	F‐Hu	14.8 (3.23)	5.81 (0.123)

*Note*: Data are presented as mean (SD). Habitats: F, Forest margin; Ho, Hollow; Hu, Hummock; O, Open expanse. Peatland sites (elevation): A, Anbei (730 m); D, Dongfanghong (1114 m); H, Hani (900 m); S, Sandaohu (597 m); Y, Yueliangwan (1000 m).

Abbreviation: WTD, water table depth.

### 2.3 Experiment design

Given the various possibilities of dormancy type in the study species, we conducted four tests and one dormancy‐breaking treatment. Firstly, to test the viability of seeds, we carried out a viability test with tetrazolium. Secondly, based on the viability test, we further cut the seeds to observe embryo morphology, to determine whether MD was present. Thirdly, we conducted an imbibition test to determine if the seed coat could not absorb water and hence limit germination, to determine the presence or absence of PY. Fourthly, before and after stratification, we carried out a germination test to determine whether PD or MPD was present in the seeds. Fifthly, combining the imbibition test, stratification treatment, and then germination test, we tried to determine the presence of PY + PD.

### 2.4 Viability test

The protocol adapted from Bourgeois et al. (2019) was used to assess viability, and 2,3,5 triphenyl tetrazolium chloride (TTC) solution was prepared with phosphate buffer. Before being dyed, seeds were soaked at 30°C for 8 h to allow complete uptake of the deionized water. Seed embryos were then exposed by a longitudinal cut and dyed with TTC at a concentration of 1% for 12 h at 30°C in darkness (20 seeds per dish). Seeds were deemed viable if their embryos were firm, red, and showed no signs of necrosis. Otherwise, they were regarded as nonviable. For *Scheuchzeria palustris* seeds, a simple visual inspection was used (Bourgeois et al., 2019). Specifically, a seed is deemed as viable if the internal color, upon cutting, appears as emerald green or dark green. Conversely, if the color is brown or black, the seed is considered as nonviable.

### 2.5 Morphological observation of embryos

Seeds were soaked in deionized water for 24 h and then cut longitudinally with a scalpel. The morphology of seed embryos was observed using stereomicroscopes (SMZ1270, Nikon, Japan). The embryo‐to‐seed ratio (E:S ratio) was calculated by dividing the embryo length by the seed length after measuring. We followed the criteria of Baskin and Baskin (2014) to tell the status of embryo, namely, differentiated, undifferentiated, and differentiated but underdeveloped. If a radicle and cotyledon(s) can be distinguished, the seed embryo was regarded as differentiated. Otherwise, it was regarded as undifferentiated. If a radicle and cotyledon(s) can be distinguished but the volume proportion of embryo‐to‐seed was no more than 0.1%, the seed embryo was considered as differentiated but underdeveloped. For the seed with embryo development and differentiation, if germination happens within 4 weeks in a germination test, it was determined as morphological dormancy; if gemination cannot happens within 4 weeks, it was determined as morphophysiological dormancy.

### 2.6 Imbibition test

To determine whether the seed coat is permeable, that is, whether there is PY, we conducted an imbibition test (Baskin & Baskin, 2004). For seeds of species weighing more than 1 mg, we used three replicates containing 20 seeds for each species, while for seeds with weight less than 1 mg, we used 50 seeds per replicate. The seeds were placed in a Petri dish with two layers of Cytiva filter paper and incubated in a growth chamber (PRX‐450C, Ningbo Saifu, China) under white light at 25°C for 72 h, seed mass was measured after 0, 24, 48, and 72 h (ISTA, 2007). During the measurement, the seeds were removed from the wet filter paper and weighed again after being wiped dry. A significant increase in seed mass indicates the presence of permeable seed coat, while little or no increase in mass indicates the presence of impermeable seed coat. To account for the different seed mass, we converted the number of seeds to 20 during data analysis. In addition, for seeds of *H. longistylum* and *V. uliginosum* with weak water permeability, we took scanning electron microscope (SU8010, Hitachi, Japan) photos to further observe seed coat thickness and the presence of an impermeable layer.

### 2.7 Dormancy breaking and germination test

A two‐way factorial experiment was employed with two factors: seven levels of dormancy breaking and 15 levels of species, with four replicates each (420 samples in total). The seven levels of dormancy breaking included: control (CK, germination percentage before stratification), dry storage (D), GA soaking (GA), cold stratification (CS), warm + cold stratification (WCS), GA soaking + cold stratification (GA + CS), GA soaking + warm + cold stratification (GA + WCS).

Dry storage involved storing the seeds in a 4°C refrigerator for 5 months. For the GA treatment, the seeds were soaked in 500 mg/L GA_3_ for 12 h after pretreatment with 4 mol/L H_2_SO_4_ for 15 min which was demonstrated to well facilitate seed absorption of GA in a pre‐experiment. CS was carried out by storing the seeds at 1°C and relative humidity (RH) 50% in darkness for 12 weeks. Variable‐temperature stratification was conducted over a total of 18 weeks to simulate the late summer and early autumn climate in the Changbai Mountains. During weeks 1–6 (warm stratification), the incubator temperature was set at 25/15°C with a 14/10 h (light/dark) and RH 50%, photoperiod was the same as above. During 7–18 weeks (cold stratification), the growth chamber temperature was set at 1°C and RH 50% to simulate the winter climate in the region. Given the snow cover in winter and early spring, 24 h in darkness was used. To ensure that the seeds remained moist, a weekly flip check was performed during stratification under weak green light.

Preliminary experiments showed that seeds of many species exhibit low or no germination percentage at a constant temperature, but the germination percentage increases with the number and range of alternating temperature (Fenner & Thompson, 2005). Pre‐experiments have also demonstrated that seeds tend to germinate much better in an alternating temperature environment (25/15°C) than in constant temperature environment (20°C). Therefore, the thermoperiod of seed incubation in this study was set at 25/15°C. After cold, warm, or variable‐temperature stratification, the seeds were incubated for 4 weeks in 12/12 h light/dark photoperiod at 25/15°C, and germination percentages were recorded. Observations were carried out on day 1, 3, 5, 7, 14, 21, and 28 after incubation, with germination determined by the emergence of the embryo through the seed coat.

### 2.8 Data acquisition for maternal environment

We used the WorldClim database (https://www.worldclim.org/data/worldclim21.html), to obtain data on annual precipitation, annual mean temperature, temperature seasonality, and precipitation seasonality for each peatland. Water table depth (WTD) for the typical habitat of each species was determined by measuring the vertical distance from peatland surface to water table level using a tape measure (Yi et al., 2024). Water pH was determined by averaging values of three repeated measurements ~20 cm apart with the portable multi‐parameter analyzer (HQ30D, Hach, USA) in the nearest surface water within the typical habitat. Both WTD and pH measurements were collected from five typical habitats for each species more than 5 m apart in the peatlands.

### 2.9 Data processing and analysis

#### 2.9.1 Data processing

The dyeing percentage of seeds was used to represent the viability of seeds, proportion of imbibed seed weight to initial seed weight was used to represent their permeability. Germination percentage was calculated as the proportion of germinated seeds to the total number of seeds in the Petri dish.

In the stratification experiment, there were 25 seeds per dish and four replicates were conducted. Although light availability was low, some seeds germinated during warm stratification in this experiment. Therefore, when counting germinated seeds, those germinated during stratification were also included.

#### 2.9.2 Statistical analysis

All statistical analyses were conducted in R v 4.2.3. Generalized linear models (GLMs) were used to analyze the effect of dormancy‐breaking treatments (explained factor) on germination (germination percentage on the 28th day as dependent factor). The significance of factors for each experiment was assessed by Wald Chi‐square statistics to the model. Duncan's test was used for multiple comparisons. The difference between germination percentage of control and initial viability was determined by Mann–Whitney *U*‐tests to determine whether the seeds were dormant or not. Principal components analysis (PCA) was used to reduce and visualize the variability in the species' seed germination response, and to determine intercorrelations among seed traits or environmental factors. The PCA was carried out with the package ‘FactoMineR’ using the variance–covariance matrix. The significance level was set to *α* = .05.

## 3 RESULTS

### 3.1 Initial seed viability

**FIGURE 2:**
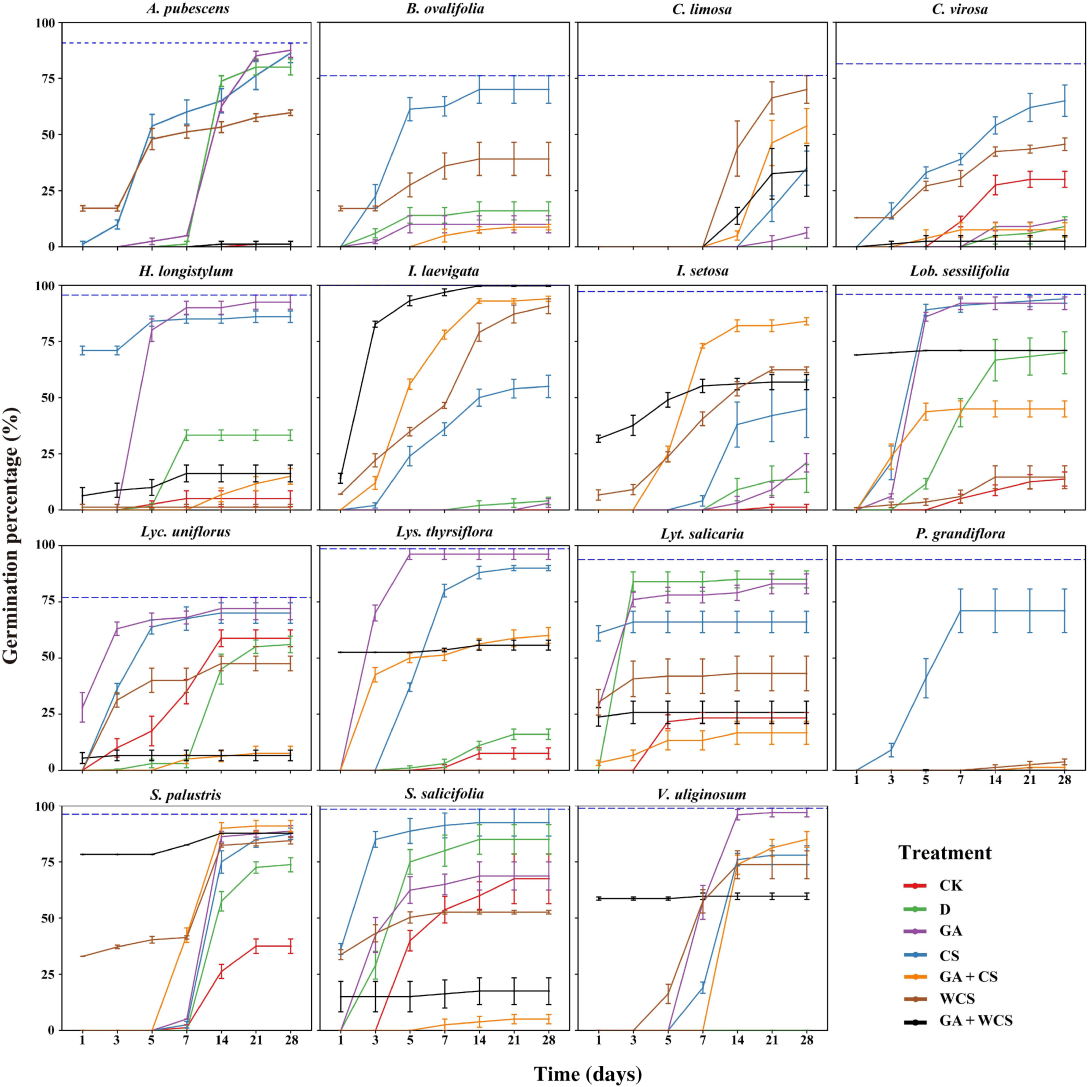
Germination percentage of study species' seeds in peatlands of the Changbai Mountains under six dormancy‐breaking treatments. CK, control; D, dry storage; GA, gibberellic acid soaking; CS, cold stratification for 12 weeks; WCS, warm stratification for 6 weeks followed by cold stratification for 12 weeks; GA + CS, gibberellic acid soaking followed by cold stratification for 12 weeks; GA + WCS, gibberellin acid soaking followed by warm stratification for 6 weeks and then cold stratification for 12 weeks (mean ± SEM, *n* = 4). The blue dashed line represents the initial seed viability (*n* = 4). Full names of the 15 species are shown in Table 1.

The initial seed viability of the 15 tested species ranged from 76.1% to 100% with an average of 89.2 ± 3.74% (mean ± SEM). *Betula ovalifolia* (Figure 2) seeds had the lowest initial viability of 76.1 ± 4.73%. The average viability of *C. limosa*, *C. virosa* and *Lyc. uniflorus* seeds ranged between 76.3% and 81.5%. All other species had viability greater than 90% (the dashed line shown in Figure 2).

### 3.2 Seed morphological observation of the embryo and imbibition

**FIGURE 3:**
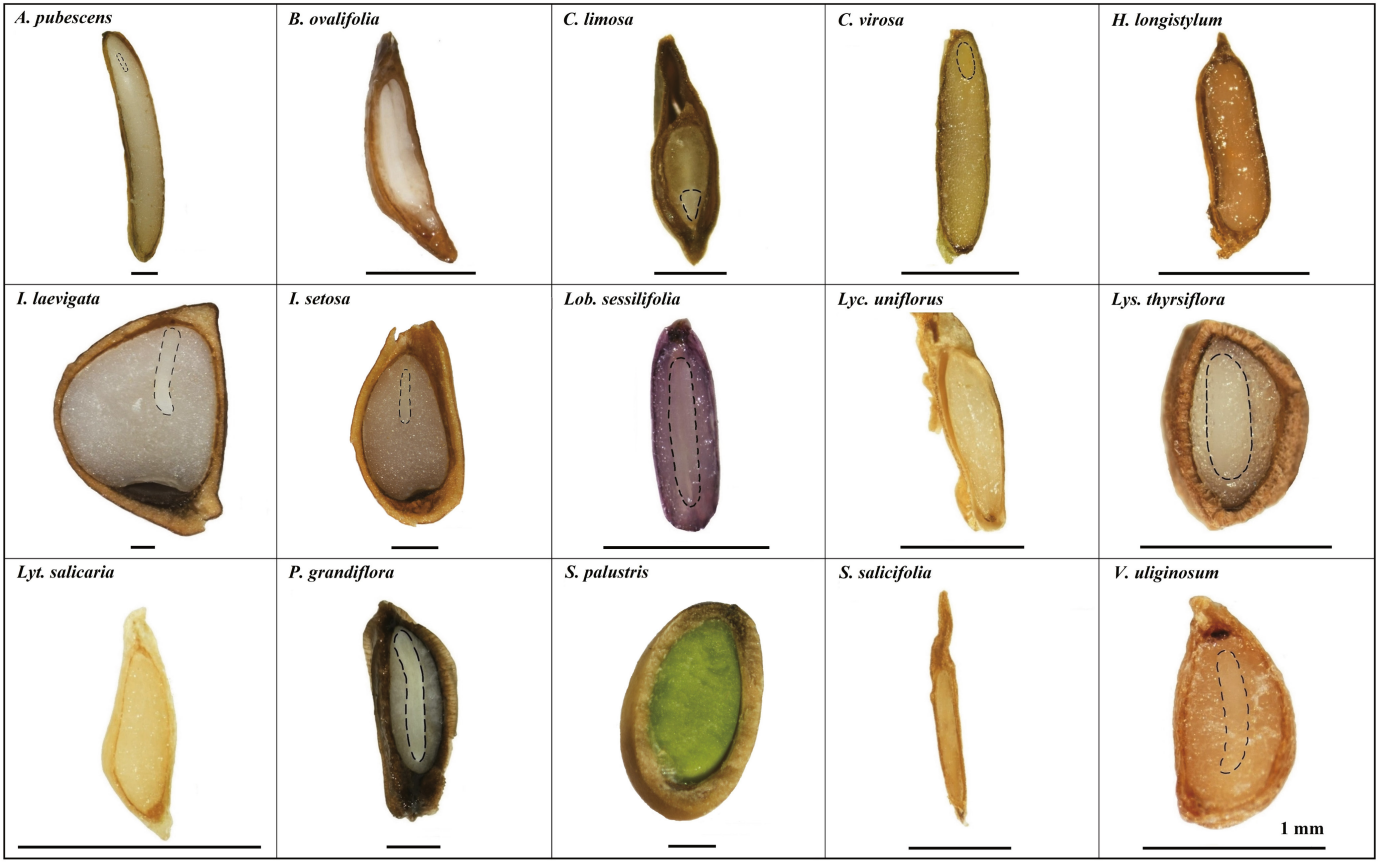
Morphological structure of seeds of study species in the Changbai Mountain peatlands. Bars, 1 mm. The dashed line indicates the outline of the embryo; for exalbuminous seeds, the embryo is not drawn separately with a dashed line.

The seeds of *A. pubescens*, *C. virosa*, *I. laevigata*, and *I. setosa* were with linear underdeveloped embryos surrounded by endosperm (with a short hypocotyl and low E:S ratio of 0.08–0.55), but the seeds of all the other tested plants (11 species) were well differentiated and fully developed at the time of collection (Figure 3).

After imbibition for 24 h, all species showed a clear increase in seed weight and then no further increase was observed thereafter (see Appendix S1). The proportion of fresh weight increase varied among the tested species, with 50%–100% for *C. virosa*, *I. laevigata*, *I. setosa* and *P. grandiflora*, over 100% for *A. pubescens*, *C. limosa* and *S. palustris*, and 0–50% for the other species. Notably, *H. longistylum* showed the lowest water imbibition capacity among the tested species. In addition, no impermeable layer was found under the observation with a scanning electron microscope (Appendix S4).

### 3.3 Effect of dormancy‐breaking treatment on seed germination

Under the control treatment, except for the two non‐dormant species (*S. salicifolia* and *Lyc. uniflorus*), the initial germination percentage of the other 13 species was low, ranging from 0 to 37.5%, with an average of 16.39 ± 2.95%, suggesting the presence of dormancy in these species. Compared with the control, dry storage improved seed germination percentage of the tested species (*χ* ^2^ = 540.2, *p* < .001), except *C. virosa* and *Lyc. uniflorus*. It even resulted in *Lyt. salicaria* seeds achieving the highest germination percentage of 85 ± 3.79% (Figure 2). Gibberellic acid (GA) soaking increased the germination of *A. pubescens*, *H. longistylum*, *Lyc. sessilifolia*, *Lys. thyriflora*, *S. palustris* and *V. uliginosum* seeds (*χ* ^2^ = 928.1, *p* < .001), with germination percentage greater than 85% (Figure 2). CS significantly increased germination percentage of all species (*χ* ^2^ = 2041.6, *p* < .001). Cold stratified seeds of *Lob. sessilifolia*, *Lys. thyriflora* and *S. salicifolia* species showed germination percentage greater than 90% while *B. ovalifolia*, *C. virosa*, *Lob. sessilifolia*, *S. salicifolia*, and *P. grandiflora* seeds achieved their highest germination percentage (Table 2, Figure 2). Varied‐temperature stratification (WCS) (*χ* ^2^ = 117.4, *p* < .001) enhanced seed germination percentage of most species, but it inhibited the germination of some species, such as *H. longistylum*, *Lyc. uniflorus*, *Lys. Thyriflora*, and *S. salicifolia*. The GA + CS treatment resulted in the highest germination percentage in *I. laevigata*, *I. setosa*, and *S. palustris* seeds (*χ* ^2^ = 724.2, *p* < .001), but it inhibited seed germination in *A. pubescens*, *C. virosa*, *Lyc. uniflorus*, *Lyt. salicaria*, and *S. salicifolia*. Surprisingly, the GA + WCS treatment had the best effect on dormancy breaking of *I. laevigata* seeds (*χ* ^2^ = 74.5, *p* < .001), with germination percentage of 100%. The effect of dormancy breaking treatment on mean germination time (MGT) is species‐specific (see Appendices S2 and S3). On average, namely, species difference being neglected, GA, CS, WCS, and GA + CS all increased seed MGT.

**TABLE 2:** Generalized linear mixed model (GLMM) fitted to the results of the effect of different dormancy‐breaking treatments on the 28th day germination percentage (GP) of 15 peatland plant species.

Species	*D*	GA	CS	WS	GA + CS	GA + WS
*Angelica pubescens*	**474.2**	**568.8**	**552.4**	**260.5**	0.12	<0.001
*Betula ovalifolia*	**7.2**	2.8	**138.2**	**43.1**	2.2	<0.001
*Carex limosa*	<0.001	0.48	**15.0**	**59.8**	**35.3**	**13.9**
*Cicuta virosa*	**14.6**	**10.7**	**40.6**	**8.1**	**16.8**	**25.0**
*Hypericum longistylum*	**44.4**	**423.6**	**363.0**	0.78	**5.5**	**7.0**
*Iris laevigata*	1.3	0.72	**243.1**	**661.2**	**710.2**	**799.5**
*Iris setosa*	2.4	**5.8**	**28.4**	**55.4**	**101.4**	**45.9**
*Lobelia sessilifolia*	**74.9**	**144.9**	**152.4**	0.018	**23.1**	**77.5**
*Lycopus uniflorus*	0.27	**6.2**	**4.5**	**4.5**	**93.4**	**96.5**
*Lysimachia thyrsiflora*	**7.0**	**762.4**	**658.8**	**5.4**	**266.8**	**224.6**
*Lythrum salicaria*	**77.1**	**72.2**	**36.9**	**7.9**	0.9	0.12
*Pedicularis grandiflora*	<0.001	<0.001	**181.0**	0.51	0.056	<0.001
*Scheuchzeria palustris*	**112.8**	**225.4**	**214.5**	**189.1**	**245.0**	**217.1**
*Spiraea salicifolia*	3.8	0.019	**7.8**	2.7	**48.6**	**31.1**
*Vaccinium uliginosum*	<0.001	**440.6**	**284.9**	**254.7**	**338.3**	**167.2**

*Note*: The significance level of experimental treatment effects on GP of the species was assessed by Wald's *χ* ^2^ statistics of generalized linear model. Significant parameters (*p* < .05) are bolded.

### 3.4 PCA analysis on seed germination response to the dormancy‐breaking treatments

**FIGURE 4:**
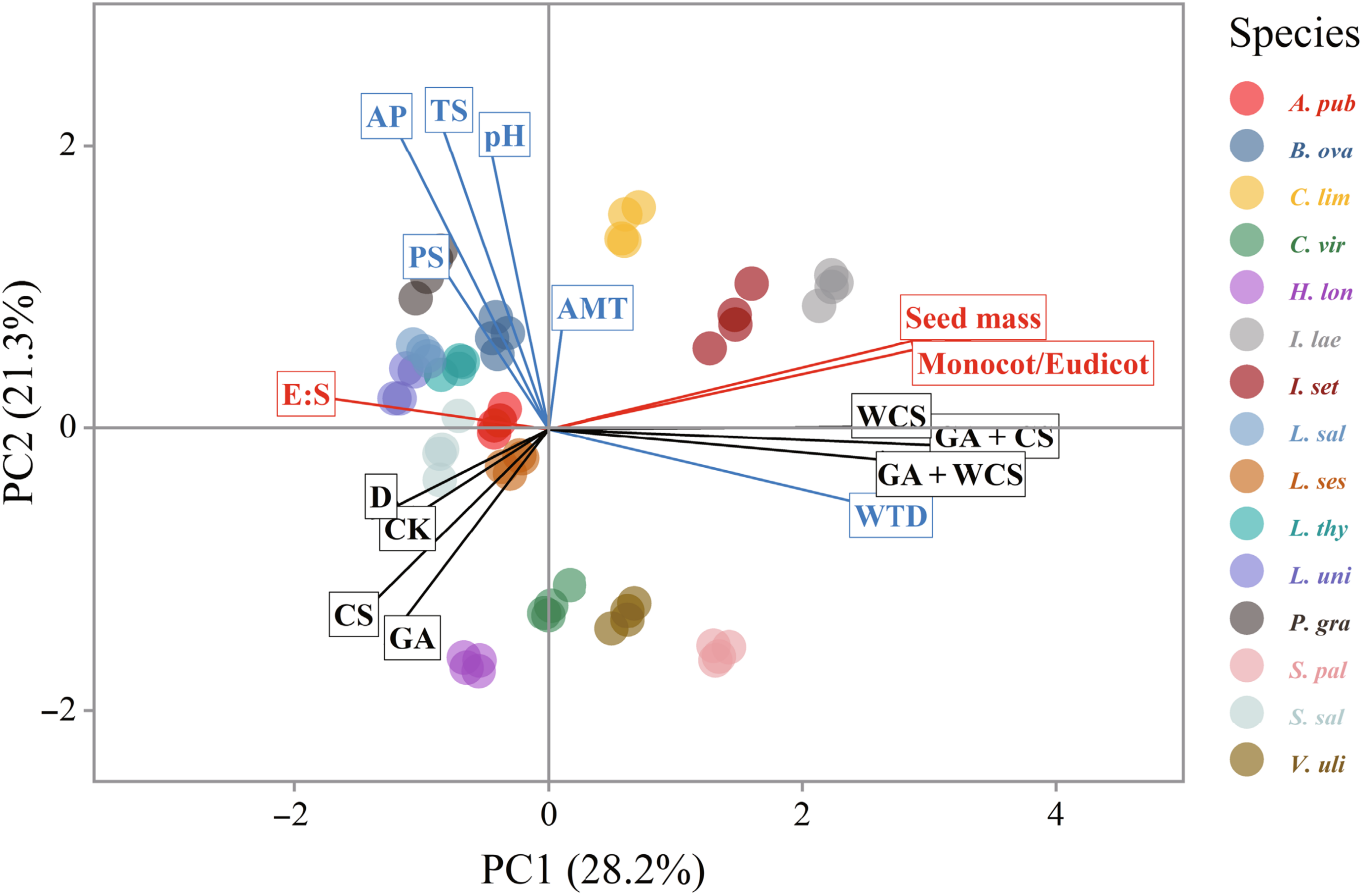
PCA analysis of study species in the peatlands of the Changbai Mountains was based on environmental factors, seed traits and germination response. The environmental factors are annual precipitation (AP), mean annual mean temperature (AMT), temperature seasonality (TS), precipitation seasonality (PS), water table depth (WTD) and pH, represented by blue arrows and fonts. Seed traits include embryo‐to‐seed ratio (E:S ratio), seed mass and monocot/eudicot divergence, and represented by red arrows and fonts. Axis 1 corresponded to seed traits, explained 28.2%; axis 2 corresponds to environmental factors with 21.3% explanation. CK, control; D, dry storage; GA, gibberellin acid soaking; CS, cold stratification for 12 weeks; WCS, warm stratification for 6 weeks followed by cold stratification for 12 weeks; GA + CS: gibberellin acid soaking followed by cold stratification for 12 weeks; GA + WCS, gibberellin acid soaking followed by warm stratification for 6 weeks and then cold stratification for 12 weeks (mean ± SEM, *n* = 4). Full names of the 15 species are given in Table 1.

In Figure 4, the first three PCA axes explain 63.25% of the variance. Axis 1 (28.17% of variance) was positively correlated with the seed traits, including seed size, E:S ratio and monocot/eudicot divergence. This indicated that there was a differentiation in seed germination between large and small‐seeded species, long‐ and short‐embryo species, and monocotyledonous and eudicotyledonous species. Seeds of monocotyledonous, embryo‐short/embryo‐underdeveloped, and large‐seeded species (e.g. *S. palustris*, *I. laevigata*, *I. setosa* and *C. limosa*) required more complex and special treatments to germinate, whereas those of eudicotyledonous, endosperm‐less/embryo fully developed and small‐seeded species seemed to easily germinate under natural conditions. On the other hand, axis 2 (21.34% of variance) was mainly positively correlated with annual precipitation, annual mean temperature, temperature seasonality, precipitation seasonality, and pH, but negatively correlated with WTD, indicating that germination percentage of the tested plants was closely related to these ecological/environmental factors. Specifically, deeper water table, less mean annual precipitation, strong seasonality, lower mean annual temperature, and lower pH were all associated with high seed germinability.

## 4 DISCUSSION

The purpose of this study was to investigate seed dormancy type and germination characteristics of peatland plants in temperate Asia with terrestrial monsoon climate. In the experiment, we systematically used a series of dormancy‐breaking means. We expected that PD was the primary dormancy type for the tested species from continental monsoon climate with strong seasonality of temperature and precipitation. Our results demonstrated that nearly 90% of species had dormancy; 60% of dormant showed PD; CS, the most efficient way to break seed dormancy, and both seed traits (e.g. embryo‐to‐seed ratio and seed size) and abiotic environmental factors (e.g. pH and temperature seasonality) affected seed germination.

### 4.1 Dormancy types of peatland plant seeds in the Changbai Mountains

A non‐parametric significance test including both initial viability and initial germination percentage showed that dormancy was present in 13 out of 15 plant seeds, except *Lyc. uniflorus* and *S. salicifolia* seeds. Notwithstanding this, we contend that both species showed germination percentage of 58.75% and 67.5% in CK. However, after dormancy‐breaking treatment, the germination percentage and mean germination time increased.

The species with dormancy in this study can be classified into two dormancy types, MPD and PD, according to the classification system of Baskin and Baskin (2004). All the tested species showed permeable seed coat, indicating no physical dormancy (PY). The embryos of seeds in *A. pubescens*, *C. virosa*, *I. setosa*, and *I. laevigata* were underdeveloped. Treatment with GA, CS, and WCS for breaking dormancy resulted in the germination of seeds from *A. pubescens*, *C. virosa*, and *I. setosa*. These findings indicate that these three species exhibit non‐deep simple MPD. Furthermore, variable‐temperature stratification, but not GA soaking, successfully broke the dormancy of *I. laevigata*, indicating that this species has deep simple MPD. Although CS was less effective than GA treatment, it uniquely increased seed germination percentage in *P. grandiflora*, clearly indicating that this species has intermediate PD. Dry storage and GA treatment was effective in breaking the dormancy of the remaining eight species, demonstrating that these species have non‐deep PD. Therefore, the findings support our first hypothesis.

### 4.2 Effect of dormancy‐breaking treatment and seed traits on seed germination

Contrary to our second hypothesis, a strength hierarchy of dormancy breaking: CS > GA > WCS > GA + CS > D > GA + WCS was found in the study (by comparing the average germination percentage on the 28th day of 15 species among different treatments). Compared with the control, both single treatments including GA, CS, and WCS and combined treatments including GA + CS and GA + WCS, increased seed germination percentage. However, GA + WCS treatment also increased the possibility of fungal growth *A. pubescens* seeds, indicating a negative effect of the combined treatment. Varied‐temperature stratification is commonly used for the species with MPD, as it can promote morphologically and physiologically the after‐ripening of seeds to fully developed embryos (Baskin & Baskin, 2014). The mechanism behind may be that CS in varied‐temperature stratification breaks epicotyl dormancy, while warm stratification breaks radicle dormancy (Zhang, Pan, et al., 2022). However, in our experiment, this treatment inhibited seed germination in *H. longistylum*, *Lyc. uniflorus*, *Lys. thyriflora* and *S. salicifolia*, probably because of the absence of MPD or the presence of conditional dormancy (the transitional state between dormancy and nondormancy). CS treatment significantly increased germination percentage and reduced the mean germination time for all species, similar to the findings by Poschlod (1990) and Fernández‐Pascual et al. (2021). This phenomenon occurs because low temperature during winter can break dormancy, and subsequent soil warming functions as a cue to stimulate germination (Finch‐Savage & Footitt, 2017). Additionally, in this study, the GA treatment applied included sulfuric acid pretreatment, which caused some mechanical damage to the seed coat, resulting in increased permeability and removal of potential PD. Although all studied species had permeable seed coat, there were variations in their response to GA across species. This treatment reduced germination percentages of the seeds with thin seed coat such as *S. salicifolia* and *Lyt. salicaria*, but increased germination percentage of the seeds whose coat is thick and with poor permeability like *H. longistylum* and *V. uliginosum*.

### 4.3 Effect of monocot/eudicot divergence, seed size, and E: S ratio on seed germination

The results revealed that the seed size, monocot/eudicot divergence, and E:S ratio had significant effects on seed germination behavior. In particular, seed size was negatively correlated with germination percentage, which confirmed hypothesis 3. The monocotyledonous species with no embryos or undeveloped embryos in seeds, such as *I. laevigata*, *I. setosa*, *C. limosa*, and *S. palustris*. These species all producing big seeds, showed increased seed germination percentage after WCS, GA + CS, or GA + WCS. Conversely, the eudicotyledonous species with fully developed embryo or no endosperm, such as *Lob. sessilifolia*, *Lyc. uniflorus*, *Lyt. Salicaria*, and *S. salicifolia*, all producing small seeds, could germinate without dry storage or CS.

In our study, compared with the seeds of some eudicotyledonous species without endosperm, monocotyledonous seeds with endosperm require a longer time to provide nutrients to the growth of the embryo, resulting in a faster germination percentage of eudicotyledonous seeds than monocotyledonous seeds (Zhao et al., 2021). Similarly, in a study of 570 species of alpine meadows in the eastern Qinghai‐Tibet Plateau, a negative correlation between seed germination percentage and seed mass was also observed (Bu et al., 2007). This is because the seedlings produced by small seeds are smaller, and the rapid germination strategy allows them to enter the seedling growth stage earlier, giving them a shorter time to survive the fragile seedling period, which provides them with the temporal and spatial advantage over competing with seedlings produced by large seeds (Grime, 2002). Large seeds, however, have thicker seed coats, which are physically difficult for radicles to penetrate through, and their larger endosperm provides them with enough nutrients to survive adverse environmental conditions, so they germinate less and more slowly.

### 4.4 Seed germination and environmental factors

Dormancy status is strongly influenced by environmental factors such as temperature (e. g. Fernández‐Pascual et al., 2013; Finch‐Savage & Footitt, 2017; Zhang, Liu, et al., 2022), precipitation (Rosbakh et al., 2023; Zhang, Liu, et al., 2022), elevation (Chen et al., 2023; Ooi et al., 2012; Rosbakh et al., 2022), latitude (Rosbakh et al., 2023; Rubio de Casas et al., 2017; Zhang, Liu, et al., 2022), etc. Our analysis revealed that seed germination percentage was positively correlated with WTD but negatively correlated with habitat pH, annual precipitation, annual mean temperature, temperature seasonality, and precipitation seasonality, this confirms our hypothesis 4. These findings suggest that differences in dormancy are also associated with environmental change. For example, water table fluctuation may affect spore germinationability (Feng et al., 2017; Bu et al., 2017) and increase spore dormancy in *Sphagnum* (Fan et al., 2023). In our study, both WTD and pH are with small span, 5–19 cm for the former and 5.3–6.1 for the latter. Hence, we speculate that this trend would be more pronounced if more types of peatlands such as bogs and rich fens were included since the spans of both WTD and pH would be highly expanded.

Seed dormancy is an effective way to delay germination until environmental conditions become benign (Pausas et al., 2022; Zhang, Liu, et al., 2022). Rosbakh et al. (2023) showed that the annual range of temperature and precipitation seasonality had a significant effect on PD. Our study showed that seasonality seemed to lead the seeds to wait for suitable hydrothermal conditions to break dormancy and then germinate. However, in several species, release from dormancy is completed only after the seeds have been exposed to fluctuating temperatures (Chia et al., 2016). Similar to the finding of a recent study (Jiménez‐Alfaro et al., 2018), our study also demonstrated that annual mean temperature was less important than annual precipitation, and the areas with low precipitation and a deeper WTD were more suitable for seed germination.

### 4.5 Seed dormancy types and phylogeny of plants

Baskin and Baskin (2014) indicated that many species of Apiaceae have underdeveloped linear embryos surrounded by endosperm and their seeds therefore demonstrate typical MD. Morphophysiological dormancy (MPD) exists in most plants of the genus *Angelica* and the dormancy can be broken by CS (Baskin & Baskin, 2014), consistent with the results of *A. pubescens* in this study.

Mechanical resistance of the seed coat is the main cause of dormancy in *Iris* spp. (Blumenthal et al., 1986), and that dormancy in *I. setosa* is closely related to the presence of germination inhibitors in the endosperm (Lu et al., 2008). A study by Holloway (1987) on *I. setosa* showed that the seed germination percentage was the highest when GA_3_ and CS were combined used. Diao and Gao (2006) observed that GA treatment did not, but CS significantly did increase the germination percentages of *Iris* seeds, fully demonstrating the better effect of natural stratification than GA, which is supported by the results of our study.

Among the species with seed PD in this study, *Lyt. salicaria*, whose seeds were found to germinate rapidly at all six treatments, had the highest seed germination percentage after dry storage, indicating that this species has low environmental requirements for germination. High seed production, great dispersal ability due to small mass and high growth rate of seedlings (Shipley & Parent, 1991) and extremely long‐term persistent soil seed bank (>50 years) (Poschlod, 1993) are important reasons why this species has flourished in many habitats and even becomes an invasive species (Goodell & Parker, 2017). In addition, *Lobelia* seeds may exhibit MPD, MD (Baskin et al., 2020) or PD (Ronnenberg et al., 2008). This diversity of dormancy types may reflect the diverse selection pressures the plants of the genus has faced during evolution.

## 5 CONCLUSIONS

In summary, out of the 15 tested peatland species in the study, 13 species clearly showed dormancy. Among them, eight species exhibited non‐deep PD, one species showed intermediate PD, and four species demonstrated MPD, suggesting that non‐deep PD of seeds is common in the temperate Asian montane peatland ecosystem. Overall the effect hierarchy of breaking dormancy is CS > GA > WCS > GA + CS > D > GA + WCS, in which CS and GA are time‐saving treatments in breaking seed dormancy of peatland plants in the Changbai Mountains. Our study suggests that physiological dormancy of seeds is a common adaptation strategy for the temperate montane peatland plants, allowing them to cope with the strong seasonality. Both seed traits (e.g. E:S ratio and seed size) and abiotic environmental factors (e.g. WTD, pH, annual precipitation, annual mean temperature, temperature seasonality and precipitation seasonality) are related to germination behavior. Therefore, once seed dormancy being broken by low temperature in winter, the peatland plants can realize effective recruitment by seed germination and consequently seedling establishment in spring or early summer.

## AUTHOR CONTRIBUTIONS

**Jian‐Yi Wang:** Conceptualization (equal); data curation (lead); formal analysis (lead); investigation (equal); methodology (lead); visualization (lead); writing – original draft (equal); writing – review and editing (equal). **Zhao‐Jun Bu:** Conceptualization (equal); funding acquisition (lead); investigation (equal); methodology (equal); project administration (lead); writing – original draft (equal); writing – review and editing (supporting). **Peter Poschlod:** Writing – review and editing (supporting). **Shuayib Yusup:** Formal analysis (equal); writing – original draft (supporting). **Jia‐Qi Zhang:** Formal analysis (equal). **Zheng‐Xiang Zhang:** Conceptualization (equal); writing – review and editing (supporting).

## FUNDING INFORMATION

The National Nature Science Foundation of China (Nos. U23A2003, 42,371,050 and 41,871,046) and Jilin Provincial Science and Technology Development Project (Nos. 20210402032GH and 20230203002SF).

## CONFLICT OF INTEREST STATEMENT

The authors declare no conflict of interest.

## Supporting information


Appendix S1

Appendix S2

Appendix S3

Appendix S4

## Data Availability

The data that support the findings of this study are available in Dryad at: https://doi.org/10.5061/dryad.dz08kps4x.
