# Supplementary figures and images for: Seed dormancy types and germination response of 15 plant species in temperate montane peatlands
Source: Ecol Evol. 2024 Jul 1;14(7):e11671. doi: 10.1002/ece3.11671 (PMC11216845; doi:10.1002/ece3.11671)

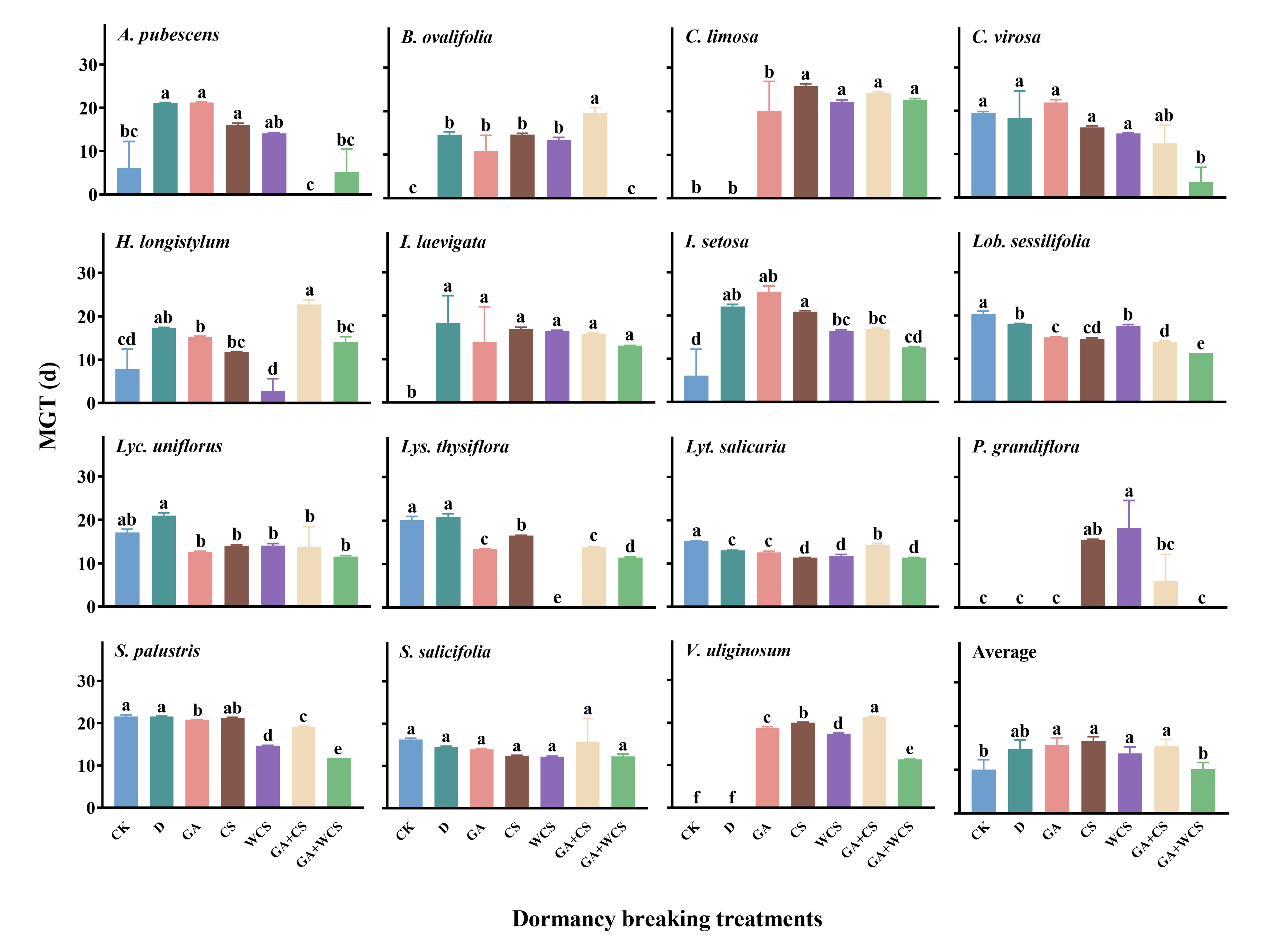

Supplement: Supplementary file 2 — Appendix S2 [file ECE3-14-e11671-s002.tif]

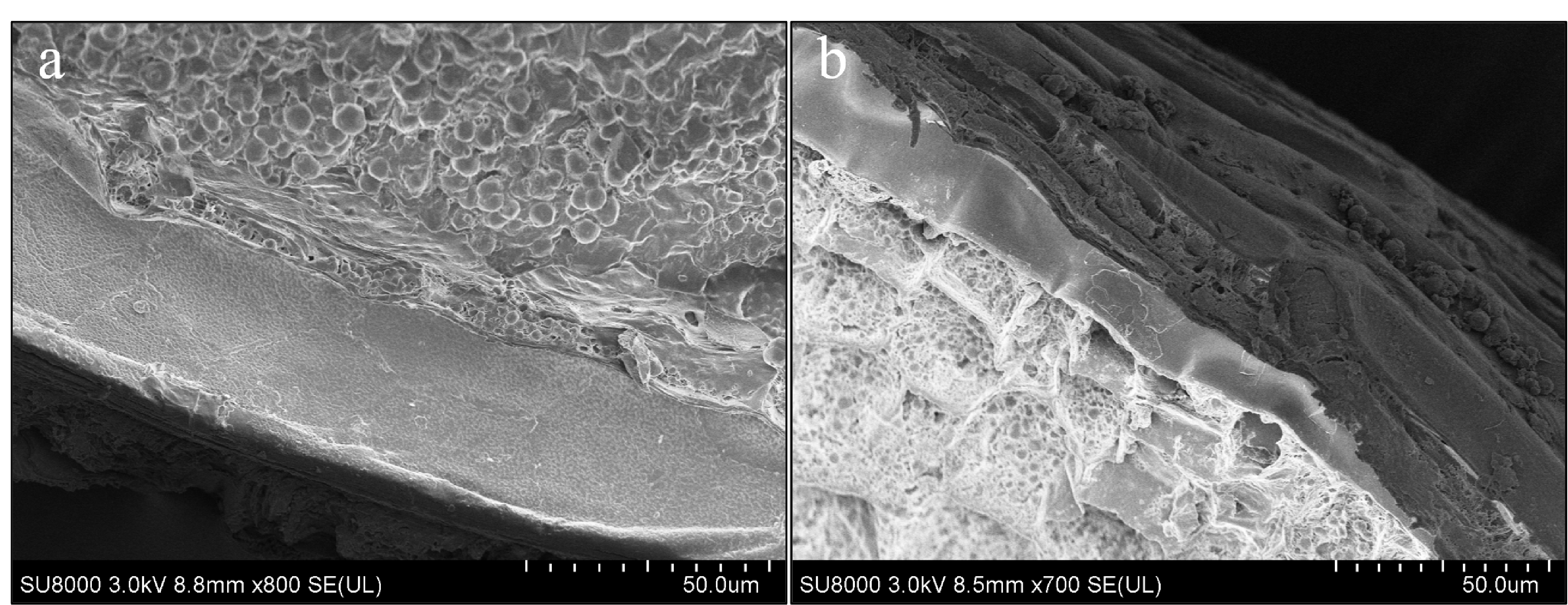

Supplement: Supplementary file 4 — Appendix S4 [file ECE3-14-e11671-s003.tif]
