# Supplementary material for: Seed dormancy types and germination response of 15 plant species in temperate montane peatlands
Source: Ecol Evol. 2024 Jul 1;14(7):e11671. doi: 10.1002/ece3.11671 (PMC11216845; doi:10.1002/ece3.11671)
Supplement: Supplementary file 3 — Appendix S3 [file ECE3-14-e11671-s001.docx]

**Appendix S3.** Generalized linear mixed model (GLMM) fitted to the results of the effect of different dormancy-breaking treatments on the 28day MGT of 15 peatland plant species.

| Species | D | GA | CS | WS | GA+CS | GA+WS |
| --- | --- | --- | --- | --- | --- | --- |
| *A. pub* | **12.0** | **12.3** | **5.3** | 3.4 | 2.0 | 0.041 |
| *B. ova* | **48.0** | **26.6** | **48.2** | **40.3** | **86.6** | <0.001 |
| *C. lim* | <0.001 | **30.7** | **50.5** | **37.3** | **44.7** | **38.7** |
| *C. vir* | 0.076 | 0.29 | 0.57 | 1.1 | 2.5 | **13.0** |
| *H. lon* | 10.0 | **6.2** | 1.7 | 2.9 | **24.8** | **4.4** |
| *I. lae* | **11.2** | **6.5** | **9.6** | **9.0** | **8.4** | **5.7** |
| *I. set* | **22.5** | **33.0** | **19.2** | **9.4** | **10.3** | 3.8 |
| *L. ses* | **29.6** | **157.7** | **178.0** | **41.0** | **227.6** | **440.7** |
| *L. uni* | 2.4 | 3.1 | 1.4 | 1.4 | 1.6 | **4.8** |
| *L. thy* | 1.5 | **115.1** | **32.5** | **1053.0** | **100.2** | **193.0** |
| *L. sal* | **71.1** | **104.8** | **226.3** | **177.2** | **11.0** | **227.9** |
| *P. gra* | <0.001 | <0.001 | **11.1** | **15.3** | 1.7 | <0.001 |
| *S. pal* | 0.0022 | **11.3** | 2.3 | **893.7** | **112.5** | **1823.4** |
| *S. sal* | 0.36 | 0.63 | 1.7 | 2.0 | 0.03 | 1.8 |
| *V. uli* | <0.001 | **8603.8** | **9817.1** | **7440.0** | **11165.2** | **3158.6** |

Note: The significance level of experimental treatment effects on MGT of the species was assessed by Wald’s χ^2^ statistics of generalized linear model. Significant parameters (*P* < 0.05) are bolded.
